# Effectiveness of a Mindfulness-Based Intervention Program for Women Family Caregivers of Older Adults

**DOI:** 10.3390/healthcare9091216

**Published:** 2021-09-15

**Authors:** Cristina Fernández-Portero, David Alarcón, Ana Gallardo-Flores, Josue G. Amián, Jose A. Sánchez-Medina

**Affiliations:** 1 Deparment of Social Antropology, Psychology and Public Health, Pablo de Olavide University, 41013 Sevilla, Spain; cbferpor@upo.es (C.F.-P.); jgarami@upo.es (J.G.A.); jasanamed@upo.es (J.A.S.-M.); 2 Deparment of Social Work and Social Services, Pablo de Olavide University, 41013 Sevilla, Spain; amgalflo@upo.es

**Keywords:** women caregivers, health, resilience, mindfulness, well-being

## Abstract

Background: The objective of this study was to analyze the effectiveness of a mindfulness-based intervention program for the promotion of well-being and health in family caregivers. Methods: The participants were 111 family women caregivers aged between 33 and 75 years old. This was a double-blinded randomized controlled trial. The mindfulness intervention program lasted 12 weeks. The experimental group underwent mindfulness and acceptance-based interventions (MABIs). The control group performed physical activity training. Results: A cross-lagged panel analysis was computed with the two waves of health, well-being, burden and resilience and age in years and intervention as predictors. The cross-lagged path model fit well *χ*^2^ (8) = 7.179, *p* = 0.51, root mean square error of approximation (RMSEA) = 0.00, comparative fit index (CFI) = 1.00, standardized root mean square residual (SRMR) = 0.05. The mindfulness intervention program was a significant predictor accounting for decreasing health problems (β = −0.292, *p* < 0.01) and burden (β = −0.190, *p* < 0.01) and increasing well-being (β = 0.107, *p* < 0.05) at post-test. Conclusions: Mindfulness-based intervention programs are effective in coping with the burden of family caregivers and, in turn, in promoting resilience, well-being and health among caregivers. Our findings encourage clinical uses of mindfulness interventions to promote health.

## 1. Introduction

In recent decades, the number of people over the age of 60 has increased worldwide, representing 8.5% of the world’s population in 2016, 617 million older people [1]. The oldest continent is Europe with more than one-fifth of the population (20.3%) over 65. Prospective studies estimate that by 2100 this figure will increase to 31.3% [2]. In Europe, of the 447 million people in 2020, 49% (218 million) were men and 51% (228 million) were women. The dependency rate was expected to reach 82.6% in 2100 compared to 54.9% on the European continent in 2019. In this context, Spain is one of the European countries that has aged the most in recent years [3,4].

Of the 46,722,980 people in Spain, 19.1% are older people, of whom 3,839,711 are men and 5,068,440 are women. Women have a higher life expectancy than men (85.7 vs. 80.4). However, their “healthy” life expectancy is shorter. Healthy life expectancy is calculated based on chronic morbidity and self-perceived health status. This indicates that although women live longer, their health is worse than that of men. In fact, 52.3% of men perceive their health as good, while only 40% of women rate their health as good or very good [5].

Given the aging of the population in Spain, the number of people who live alone and who need care has increased drastically in the last decade [5,6]. Seventy-three percent of the population over 65 years of age has difficulties performing daily life tasks due to the appearance of diseases and illness because of the aging process [6]. These health issues require care that most of the aging population prefers to receive at home [7]. The number of family caregivers has increased, representing 80% of the total care received by older adults [6].

Family care tasks tend to fall more frequently on women than men as part of their family and social role [8,9]. In fact, 75% of family caregivers of people with disabilities and 60% of caregivers of older adults in Spain are women [10,11]. The profile of family caregivers in Spain is between 45 and 65 years old; they dedicate more than 20 h a week to care tasks and tend to have emotional ties with the people receiving care [5]. Family care comes mainly from daughters who take care of their parents, followed by other relatives and/or friends. Therefore, it is necessary to study the possible consequences of family care tasks on the health and well-being of female caregivers.

Women caregivers have poorer well-being than those who do not perform caregiving tasks for their elders [12]. This deterioration in well-being among family caregivers is frequently associated with suffering from other health problems [13]. A meta-analysis found that high levels of stress and depression in female caregivers were associated with low levels of well-being [12]. Another study with 125 family caregivers, found a relationship between high levels of depression and anxiety and care tasks [13]. Other authors highlight depressive symptoms, anxiety, and sleep problems among the most common health disorders in female caregivers [14]. These authors defend the need to promote resources that increase the well-being of family caregivers and the quality of life of patients. The negative effects on the health of female caregivers are significantly maintained over time after a period of 8 years [15]. Identifying risk and protective factors that influence health and well-being in caregivers can help to prevent these negative effects.

The perception of burden can be a risk factor for the health and well-being of family caregivers [16,17,18]. Burden is defined as the caregiver’s perception of the impact that care tasks have on their health and on their social, personal, and economic life [19]. Family care increases the quality of life of older adults due to its continuity in their social environment. However, the health and well-being of the caregiver can be affected by the perception of burden, isolating him or her and occupying all his or her free time [20]. Good physical and mental training is necessary to cope with the tensions that the caregivers’ burden generates [21].

In contrast, resilience is a protection factor against caregiver burden [22,23]. Resilience is defined as the ability to overcome adversity, recover and strengthen after exposure to a traumatic psychosocial event, and is considered a coping strategy [24,25,26]. Family caregivers who have low resilience experience a higher burden when faced with the demands of the caregiver, while family caregivers with high levels of resilience experience a lower burden due to the high demand for care [27]. Therefore, promoting the resilience of family caregivers can promote their health and well-being [28].

Recent studies show the effectiveness of mindfulness-based techniques in family caregivers [29,30,31]. A meta-analysis review concluded that mindfulness techniques are being imposed on interventions based on psychosocial, psychoeducational and counseling programs [31]. To date, only small effects of cognitive-behavioral therapy (CBT) have been shown in depression and family caregiver burden. However, interventions based on mindfulness techniques (mindfulness and acceptance-based interventions—MABIs) show significant effects on the reduction of depression and the burden of family caregivers [31]. There are different types of mindfulness-based interventions, such as interventions focused on stress reduction (MBSR), mindfulness-based cognitive therapies (MBCT), acceptance and commitment therapies (ACT) or dialectical-behavioral therapy (DBT). All of them focus on mindfulness but differ in the duration and practice of meditation and the way teaching behavior changes strategies. The study concludes that, in general, mindfulness-based intervention programs have a large effect in reducing the levels of depression in family caregivers and a moderate effect in reducing burden. However, as it is a novel field of intervention, it is necessary to continue investigating its effects on health and the burden of family caregivers [31].

The objective of this research is to analyze the effectiveness of a mindfulness-based intervention program for the promotion of well-being and health among female family caregivers. We hypothesize that the women caregivers who participated in the mindfulness intervention program would show a decrease in their perceived burden and an increase in their resilience, while the mindfulness intervention program would enhance their health and well-being.

## 2. Materials and Methods

### 2.1. Participants

The participants of the present study were 111 women aged between 33 and 75 years old (mean = 52.08 and SD = 9.34). All of them were family women caregivers who spent at least eight hours a day caring for a family member either in the caregiver’s own home or in the older adults dependent’s home. Significant differences by age were observed between the control group (CG) and experimental group (EG), *t* = 2.251, *p* < 0.05. Hence, to control for these differences between groups, age in years has been used as a covariate variable in the remaining analysis. No differences were observed between the groups by educational level (*χ*^2^ = 2.523, *p* > 0.05), and most of the family caregivers had completed only primary education. Regarding whether they lived alone or accompanied, 97.7% lived in the company of other people, and no differences were observed between the control and experimental groups (*χ*^2^ = 0.486, *p* > 0.05).

### 2.2. Instruments

The instruments for data collection were the Satisfaction with Life Scale, the Goldberg Health Questionnaire, the Connor–Davidson Resilience Scale and the Zarit Caregiver Burden Interview. The self-reported measures were collected at pre-test (Time 1, T1) and post-test (Time 2, T2).

The Satisfaction with Life Scale (SWLS) [32] measures the well-being of people with their lives. It is a Likert scale with seven Likert-type response options ranging from 1 (Strongly disagree) to 7 (Strongly agree) to higher scores higher well-being. The reliability of the scale was high in T1 (*α* = 0.873) and T2 (*α* = 0.858).

The Goldberg Health Questionnaire (GHQ-12) [33] measures psychological distress and detects changes in the psychological functioning of the person. The abbreviated version of 12 items was selected for this study with Likert-type response options ranging from 1 to 4; the more positive the response is, the more it tends to 1. The reliability of the scale was high in T1 (*α* = 0.855) and T2 (*α* = 0.863).

The Connor–Davidson Resilience Scale (CD-RISC) [34] measures resilience on a scale of 25 items on a scale of 0–4, where 0 = “has not been true at all” and 4 = “true almost forever”. The total scores range between 0 and 100; higher scores indicate greater resilience. The reliability of the scale was high at T1, with a Cronbach’s *α* of 0.905, and T2 (*α* = 0.905).

The Zarit Caregiver Burden Interview *(ZBI)* [35] measures the degree of burden of caregivers on a 22-item Likert-type scale with response options ranging from 1 (never) to 5 (almost always). The response is more positive and implies less burden the more it tends to 1. The scale had a high reliability in T1 (*α* = 0.909) and T2 (*α* = 0.907).

### 2.3. Procedure

**Figure 1:**
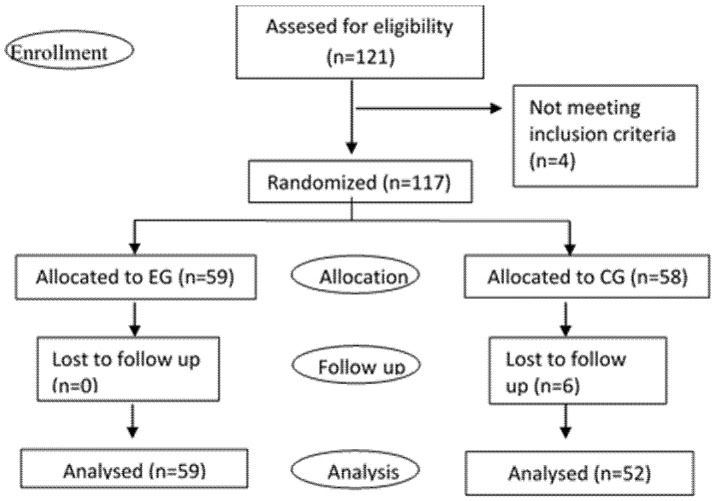
Consort diagram. Note: EG, experimental group; CG, control group.

This is a double-blinded randomized controlled trial. Family women caregivers were recruited while attending a call by the town hall to participate in a formative course. The inclusion criterion was a woman taking care of a family member over 60 years old. The number of participants was 121, but only 117 met the inclusion criteria for eligibility (four participants were men). The participants were included in the experimental or control group by random number generation by researchers not involved in the enrollment of participants, see Figure 1.

The experimental and control intervention programs lasted 12 weeks, with one session per week. Data were collected the first week before the intervention program (pre-test) and the week after the program (post-test). All participants began the intervention programs at the same time in small groups of 15 participants.

The experimental group underwent mindfulness and acceptance-based interventions (MABIs) among all the sessions, and the exercises focused on mindfulness practices and attempted to raise present awareness and acceptance, combined with yoga, breathing relaxation and meditation based on similar studies [36,37,38]. In each session, there was a particular meditation objective with a combination of mindfulness and acceptance exercises, as described in Table 1. In the mindfulness and acceptance-based intervention program, all the techniques were used with the aim of deepening awareness, self-compassion, and self-knowledge. In the final session, working groups held among the participants to evaluate the knowledge acquired during the intervention and to analyze the applications in their daily life. The structure of each MABI training session was: (1) check-in and reflecting on personal practice of previous week working activity for 10 min, (2) explaining and practicing new exercise and meditation for 40 min, and (3) debriefing the meditation experience for 10 min. The trainer of the EG was an expert in mindfulness.

On the other hand, the CG performed physical activity training under an expert trainer during the 12 weeks of the mindfulness intervention program. The structure of each physical training session was: (1) warming ups for 10 min, (2) endurance and resistance exercises for 40 min, and (3) stretching exercises for 10 min. The trainer of the CG was an expert in physical training.

### 2.4. Data Analysis

Statistical analyses were performed using SPSS version 23, employing a statistical significance at α = 0.05. Descriptive analyses were used to describe the sample characteristics (i.e., sociodemographic). Repeated-measures ANOVA F-tests were used to assess time differences by intervention group. Pearson correlation analyses were performed to evaluate scales measures associations. A cross-lagged panel analysis was computed to examine associations between pre and post intervention changes in the psychological variables employing Mplus version 8 [39].

### 2.5. Ethical Considerations

The study was conducted according to the guidelines of the Declaration of Helsinki, Voluntary informed consent was requested. Participants were informed about the aims of the research and the right to withdraw without penalty if they wished. Questionnaires were registered with strict confidentiality.

## 3. Results

To assess the effects of the mindfulness intervention program, a repeated-measures ANOVA was performed by time (T1 and T2) and group (EG and CG) with age in years as a covariate (see Table 2). There were non-significant differences between the experimental and control groups at T1 on any of the dependent variables: health, well-being, resilience, and burden. Thus, the experimental and control groups were equivalent on the dependent measures at the pre-test.

However, there were significant interaction effects between time and group on the psychological measures when considering the change between T1 and T2. There was a significant interaction effect between time and group on the health measure, F (1109) = 27.849, *p* = 0.000, ηp^2^ = 0.204. Bonferroni-adjusted post hoc tests revealed that the participant’s health improved significantly between T1 and T2 in the experimental group (*p* < 0.001), whereas there were non-significant differences in the control group (*p* = 0.148). There was a significant interaction effect between time and group on the well-being measure, F (1109) = 4.716, *p* = 0.032, ηp^2^ = 0.041. The differences by group and time show that well-being increased significantly in the EG (*p* = 0.001), but there were non-significant differences by time in the CG (*p* = 0.707). The interaction effect of time and group on burden was statistically significant, F (1109) =9.469, *p* < 0.003, ηp^2^ = 0.080. The differences show that the burden level decreased in the EG (*p* = 0.003) and increased in the CG (*p* = 0.019). There was a significant interaction effect of time and group on the resilience measure, F (1109) = 6.599, *p* = 0.012, ηp^2^ = 0.057. The differences by group and time showed that resilience decreased significantly in the CG (*p* = 0.01), but there were non-significant differences by time in the EG (*p* = 0.568). In summary, a significant intervention program effect was observed in the psychological measures of the EG, improving their health, well-being, and burden, whereas a decrease in resilience was observed in the CG over time.

A Pearson correlation analysis showed that there were no significant correlations between the age of the participants and the psychological variables observed at T1 and T2, see Table 3. The participant’s health problems were negatively correlated with well-being at T1 and at T2. Problems of health and burden were positively correlated at T1 and at T2. Problems of health were also negatively correlated with resilience at T1 and at T2. Well-being was negatively correlated with burden at T1 and burden at T2. Well-being was positively correlated with resilience at T1 and at T2. Burden was negatively correlated with resilience at T1 and at T2 (*r* = −0.248, *p* < 0.001). Table 4 shows high significant positive correlations within measures between T1 and T2.

**Figure 2:**
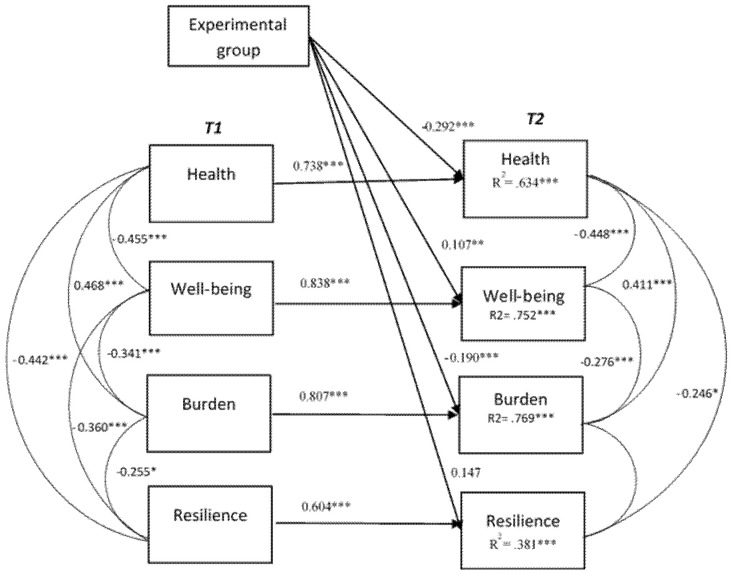
Cross-lagged relationships between health, well-being, burden and resilience at pre-test (T1) and post-test (T2). Model fit: *χ*^2^ (8) = 7.179, *p* = 0.51, RMSEA = 0.00, CFI = 1.00, SRMR = 0.05. * Correlation is significant at level 0.05; ** Correlation is significant at level 0.01; *** Correlation is significant at level 0.001.

To explain the relationships in time between the psychological variables, a cross-lagged panel analysis was computed with the two waves of the four measures and age in years and intervention as predictors. The cross-lagged path model fit well *χ*^2^ (8) = 7.179, *p* = 0.51, RMSEA = 0.00, CFI = 1.00, SRMR = 0.05. Figure 2 shows the cross-lagged model with the correlations between the variables at T1 and T2 and the percentage of explained variance of each of the variables at T2. The model explains 63% of the variance in health problems at T2, 75% of the explained variance in well-being at T2, 76% of the explained variance in burden at T2 and 38% of the explained variance in resilience at T2.

As expected, see Table 5, we found evidence for longitudinal associations between the autoregressive paths from T1 to T2; thus, the baseline measure at pre-test was a significant predictor of the post-test score of the same measure (β = 0.604 to 0.838). Likewise, for each measure, higher scores at T1 predicted higher scores at T2. The cross-lagged paths between different measures were non-significant (*p* > 0.05). Controlling for the baseline at T1, age in years was a significant predictor for wellbeing at T2 (β = 0.097) and burden at T2 (β = 0.147). Older family caregivers were likely to increase their post-test wellbeing and decrease their post-test burden. Furthermore, when controlling for the baseline at T1 and age, the mindfulness intervention program was a significant predictor accounting for decreasing health problems at T2 (β = −0.292, *p* < 0.01), increasing well-being at T2 (β = 0.107, *p* < 0.05), and decreasing burden (β = −0.190, *p* < 0.01). However, the intervention group was marginally significant in increasing resilience at T2 (β = 0.147, *p* = 0.051).

## 4. Discussion

The increase in the older population living at home has implied an increase in the need for family caregivers to help them in their daily activities [5]. In Spain, this task of family care of older adults is usually performed by women; however, performing these daily care tasks often leads to health-related consequences among the family caregivers. In this study, the main objective was to analyze the effectiveness of a mindfulness-based intervention program for the promotion of well-being and health among female family caregivers. The results indicated a significant effect of the mindfulness intervention program in improving health and well-being and reducing burden. The enhancement of the family caregiver’s well-being in the experimental group indicated that the mindfulness (MABIs)-based intervention program helped caregivers face care tasks, reducing health problems [40].

Our data support other findings who found that health problems are associated with low levels of well-being in family caregivers [13]. The increased well-being of family caregivers after the mindfulness intervention program indicates that mindfulness-based techniques promote the development of strategies and resources to cope with the negative effects of care tasks on their well-being and, in turn, on their health.

The effectiveness of the mindfulness-based intervention program was also observed in reducing the perception of burden in the experimental group [31]. Our data support this statement by observing a decrease in the burden of family caregivers between T1 and T2. The perception of burden could affect the perception of health and well-being of family caregivers [20]. Our data support this finding: there was a positive correlation between health problems and burden; that is, as the burden increased, the caregivers presented more health problems. Therefore, the mindfulness intervention program of our study supports the idea that good physical and mental preparation is needed to manage the burden and improve the well-being of caregivers [21].

However, in our study, the mindfulness intervention program did not significantly increase the resilience in the EG between T1 and T2. There was a significant decline in caregiver resilience in the CG. The mindfulness intervention program could help family caregivers rehearse their resilience [28]. Our results show that resilience promotes the health and well-being of family caregivers.

The cross-lagged analyses show that after controlling for age and the baseline measures, the mindfulness-based intervention program enhanced the health, well-being and burden of family caregivers while protecting the decline in their resilience. As previous meta-analyses show, mindfulness intervention techniques are an effective, useful, and reliable resource to improve the health and well-being of family caregivers [31]. Furthermore, daily mindfulness practices have positive effects on the well-being and health of family caregivers in the long term [38].

Among the limitations of our study was the two-point pre-post experimental design, and more previous and follow-up observation times should be included to account for the long-term effects of the mindfulness intervention on the health and well-being of family caregivers [41,42]. A further limitation of the current study is that the differential effect of a mindfulness intervention program and a physical training program was analyzed. Although previous studies have shown the psychological benefits of physical training in caregivers [43,44]; in the present study non-significant effects of the physical training program were observed in the control group. This may be because only a 1-hour session per week of physical training was performed while in the experimental group in addition to the weekly mindfulness session; and participants were encouraged to perform at home the activity practiced in weekly mindfulness session. Thus, the effect of the mindfulness training may be due to both the group sessions and the daily personal practice throughout the week. It would also be interesting to determine whether the results would differ if the control group were a daily active group, which in turn would allow future research on the effectiveness of mindfulness intervention programs [31].

## 5. Conclusions

Previous studies require further evidence of the effectiveness of mindfulness-based intervention programs on family caregivers’ health [14,31]. This study shows evidence of the effects of mindfulness intervention programs with family caregivers on their health and well-being. These findings support the need for intervention policies that promote resources to improve well-being and reduce the burden on family caregivers.

## Figures and Tables

**Table 1:** Mindfulness and acceptance-based intervention program sessions.

Sessions	Objective
S1: Raisin Exercise	Focus on the present
S2: Body Scan	Focus on the body
S3: Mindful Movement	Centering the mind through the body movements
S4: Mindfulness of the breath, sounds, and thoughts	Combining breathing and displacement in space
S5: Acceptance of thoughts and feelings exercise	Learning to know yourself
S6: Mindful Seeing	Encourage contact with the environment
S7: Lake meditation	Self-compassion and acceptance and taking care of yourself
S8: Acceptance of Social Anxiety	Connecting self-knowledge with relationships with others
S9: Lovingkindness Meditation	Compassion and acceptance to the others
S10: Mountain Meditation	Raising self-esteem
S11: Turning Toward Meditation	Dealing with difficult emotions and/or physical pain
S12: Silent meditation	Evaluation of acquired knowledge and application to daily life

**Table 2:** Descriptive analysis of health, well-being, resilience, burden and resilience in Time 1 and Time 2 by experimental group (EG) and control group (CG).

	Pre-Test (T1)		Post-Test (T2)				
	EG (*n* = 59)	CG (*n* = 52)	EG (*n* = 59)	CG (*n* = 52)	F	*p*	Eta
	Mean (SD)		Mean (SD)				
Health	2.21 (0.52)	2.06 (0.47)	1.94 (0.45)	2.12 (0.48)	27.849	0.000	0.204
Well-being	3.48 (0.98)	3.85 (1.07)	3.70 (0.94)	3.85 (1.07)	4.716	0.032	0.041
Burden	2.33 (0.64)	2.25 (0.68)	2.22 (0.63)	2.34 (0.66)	9.469	0.003	0.080
Resilience	3.65 (0.56)	3.79 (0.64)	3.70 (0.54)	3.56 (0.78)	6.599	0.012	0.057

Note: F report interaction effect on health and psychological measures.

**Table 3:** Analysis of correlations between age, health, well-being, overload and resilience in pre-test (T1) and post-test (T2).

	1	2	3	4	5
1. Age	1	0.127	0.052	0.001	−0.189
2. Health	0.086	1	−0.451 **	0.454 **	−0.277 **
3. Well−being	−0.050	−0.428 **	1	−0.215 *	0.260 **
4. Burden	0.077	0.504 **	−0.336 **	1	−0.249 *
5. Resilience	−0.038	−0.451 **	0.321 **	−0.280 **	1

Note: Time 1 correlations are in the lower part and the Time 2 correlations are in the upper right part. ** Correlation is significant at level 0.01; * Correlation is significant at level 0.05.

**Table 4:** Analysis of correlations between age, health, well-being, overload and resilience between pre-test (T1) and post-test (T2).

	Health T2	Well-Being T2	Burden T2	Resilience T2
Health T1	0.612 **	−0.372 **	0.444 **	−0.338 **
Well-being T1	−0.300 **	0.823 **	−0.319 **	0.260 **
Burden T1	0.409 **	−0.277 **	0.857 **	−0.248 *
Resilience T1	−0.268 **	0.317 **	−0.227 *	0.767 **

** Correlation is significant at level 0.01; * Correlation is significant at level 0.05.

**Table 5:** Directional path in the longitudinal panel model to examine associations between pre-test (T1) and post-test (T2) measures.

	Dependent Variables											
	Health T2			Well-Being T2			Burden T2			Resilience T2		
	b	SE	β	b	SE	β	b	SE	β	b	SE	β
Age	0.001	0.003	0.015	0.011	0.005	0.097 **	0.011	0.004	−0.147 **	−0.008	0.007	−0.112
EG	−0.286	0.055	−0.292 ***	0.218	0.094	0.107 **	0.254	0.065	−0.190 ***	0.198	0.106	0.147
Health T1	0.721	0.073	0.738 ***	0.004	0.117	0.002	0.118	0.108	0.088	0.114	0.120	0.085
Well-being T1	0.000	0.031	−0.001	0.826	0.067	0.838 ***	−0.008	0.036	−0.013	0.032	0.050	0.049
Burden T1	−0.013	0.045	−0.017	0.012	0.083	0.008	0.813	0.057	0.807 ***	0.027	0.094	0.026
Resilience T1	−0.015	0.056	−0.019	0.100	0.084	0.059	0.024	0.054	0.022	0.672	0.110	0.604 ***

Note: EG, Experimental group. * Significant at level 0.05; ** Significant at level 0.01; *** Significant at level 0.001.

## Data Availability

The data presented in this study are available on request from the corresponding author.
